# Perception of laying hen farmers, poultry veterinarians, and poultry experts regarding sensor-based continuous monitoring of laying hen health and welfare

**DOI:** 10.1016/j.psj.2023.102581

**Published:** 2023-02-13

**Authors:** Lara A. van Veen, Anna C.M. van den Oever, Bas Kemp, Henry van den Brand

**Affiliations:** ⁎ Vencomatic Group, 5520 AD Eersel, the Netherlands; † Adaptation Physiology Group, Wageningen University & Research, 6700 AH Wageningen, the Netherlands

**Keywords:** laying hen, perception, health, welfare, monitoring technique

## Abstract

Daily farm management practices play an essential role in determining and steering health, welfare and productivity of laying hen flocks. Optimal management requires expertise of farmers and coworkers, especially when hens are kept in complex, large-scale aviary systems. Relatively little sensor-based support is available to farmers, even though numerous research groups are working on developing technologies to continuously detect deviations in layer health and welfare. A survey with laying hen farmers, poultry veterinarians and poultry experts from Western Europe and Canada was conducted to identify and prioritize indicators of optimal and suboptimal laying hen health and welfare in commercial farms. The status-quo of sensor technology and the advantages, wishes, and concerns regarding sensors were additionally assessed to contribute to the future development of a predictive monitoring tool that continuously monitors laying hen health and welfare. A total of 45 stakeholders were interviewed, of which 41 filled in an online questionnaire. Although the prioritization of indicators differed between stakeholders, the majority identified the use of feed and water intake, egg production and quality, sound, activity, and movement of hens as important indicators to assess health and welfare. Currently collected (sensor) data were not used to their full potential, and stakeholders missed the integration and storage of data into one monitoring system with easy visualization tools. Most interest was observed in the use of cameras and microphones to detect deviations in health and welfare at an early stage, to reduce subjectivity of the assessment and to gain more knowledge on layer behavior. It can be concluded that these results could steer research efforts towards the development of continuous monitoring techniques, and enhance their adaptability and acceptability by stakeholders.

## INTRODUCTION

In the last few decades, the egg industry has undergone major changes due to ongoing transition from conventional cage housing toward cage-free alternative housing systems for laying hens. Compared to conventional cages, cage-free systems provide the layers with more space and freedom to express natural behaviors (Mench and Rodenburg, 2018). Concerns have been raised about increased mortality of birds in cage-free production. Schuck-Paim et al. (2021) however showed that mortality rate depends on a farmer's years of experience with the indoor cage-free system rather than on the type of housing system. Due to the complex 3-dimensional nature of aviary systems, the required management level of the farmer is high. At the same time, the average flock size has increased due to demands for both increased production and reduced environmental impact (Place). These developments have put increasing pressure on daily farm management practices in monitoring and safeguarding flock health and welfare.

Real-time sensor-based data on laying hens could support daily farm management and facilitate early recognition of decreased hen health and welfare, by providing continuous insight into the flock's status. Sensor technology is in this perspective defined as “technology to monitor laying hens, their products (eggs) and the farming environment, in order to aid farm management during the production phase, through supplying the farmer with relevant information on which to base management decisions, or by activating automated control systems.” Olejnik et al. (2022) identified 5 main areas in which tools and technologies could provide an opportunity to support farm management, namely housing and microclimate control, weight monitoring, sound analysis, locomotion and activity tracking, and disease detection and hygiene maintenance aspects. An increasing number of studies focus on the potential use of technologies in the poultry sector. However, these studies are often performed in controlled conditions rather than on large-scale poultry farms, and predominantly with broilers rather than laying hens (Rowe et al., 2019). Although these research efforts have led to the development of prototype sensors, commercialized sensor technologies are only marginally available to support poultry farm management. Commercial technologies are limited to control of the feed, water, climate and light inside the poultry house. Examples exist of automated animal weighing, activity tracking and flock distribution monitoring, and noise level monitoring.

Reasons that sensor-based monitoring techniques are marginally used to detect health and welfare problems on laying hen farms are diverse. First, there is no universal overview of current health and welfare problems that laying hens are facing. The prioritization of problems might be driven by fluctuating public concern and political interest, and the underlying criteria to rank health and welfare issues affect their prioritization. Poultry experts seem to prioritize various welfare issues differently when asked to rank them according to their prevalence, occurrence, and duration. A recent study assessed the opinion of UK-based poultry experts on the prioritization of animal welfare issues to identify key research areas. Keel bone problems on one hand received high priority as a welfare issue due to high on-farm prevalence (Rioja-Lang et al., 2020). Untreated outbreaks of feather pecking (**FP**) or cannibalism on the other hand were considered as important due to their severity and long duration. Unambiguous prioritization of key problems in the poultry sector can aid in choosing the development of monitoring techniques with the greatest added value for commercial farms.

Second, the acceptability of monitoring techniques by stakeholders in the poultry sector is not taken into account during technique development. Hubbard and Scott (2011) suggested that the acceptability of monitoring techniques could be improved when similar animal-based measures are used as those that are currently aiding farmers and other poultry experts in on-farm health and welfare assessment. Local farm conditions are thought to influence the perceived usefulness of welfare indicators as decision support tool by farmers (Vaarst, 2003).

Third, the accuracy and validity of monitoring techniques aiming at improving animal welfare are unknown. Several publications aimed to detect defined health and welfare problems by using one nonspecific indicator or indicators with ambivalent meanings that do not reliably indicate layer health and welfare in commercial settings. Integration of nonspecific and ambivalent indicators into decision tools raises concerns for false-positive alarms (Stachowicz and Umstätter, 2021).

In summary, several aspects can explain why sensor-based monitoring techniques are currently scarcely used in laying hen farms and consequently, more information is needed about the aspects mentioned above. This paper presents the current thinking of representatives from the poultry sector regarding the current and future monitoring of laying hen health and welfare. Interviews were conducted with stakeholders in the European and Canadian egg sector to identify and prioritize laying hen health and welfare problems, including their causes and (online measurable) indicators in commercial aviary systems. Moreover, the status-quo of sensor technology and the advantages, wishes, and concerns were assessed to enable future development of a predictive monitoring tool that continuously monitors animal well-being.

## MATERIALS AND METHODS

### Participant Selection

A multistakeholder approach was used to gain insight into the current thinking of laying hen farmers, poultry veterinarians and poultry experts regarding current and potential future use of sensors on poultry farms. Stakeholders worked with aviary laying hen systems in Northwestern Europe, including the Netherlands, Belgium, and Germany, where the majority of commercial laying hens are housed in these systems (Schuck-Paim). The scope was broadened toward Canada, in which the egg production sector is currently undergoing a large-scale transition from cage housing toward enriched cages and to a lower extent toward aviaries (Turner et al., 2022).

Laying hen farmers were recruited if they had a minimum of 3 yr of relevant work experience with aviary systems and carried the main responsibility for the farm and animal management. The farmers were also required to have a daily presence inside the poultry house. The minimum flock size was 3,000 hens in an aviary system in Northwestern Europe. Veterinarians and other experts were specialized in poultry, preferably focusing on laying hens, with a minimum of 3 yr of work experience in Northwestern Europe or Canada. Veterinarians were included if they worked either at a veterinary practice or at an animal health service with veterinary laboratories. The project additionally used a purposive heterogeneous sampling approach (Robinson, 2014) to create maximum heterogeneity among the homogeneous stakeholder groups. For example, stakeholders were considered for participation across a range of ages, geographical locations, and genders.

Farmers, veterinarians and experts were found through the authors’ network, through articles in print and online trade magazines and through the websites of veterinarian practices. Participants were recruited by direct contact via email, followed by a phone call, in which the study aims were described. Through a “snowball-sampling method,” potential candidates were asked to send the invitation to other poultry farmers, veterinarians or experts who met our selection criteria. Ultimately, 20 laying hen farmers, 9 specialized poultry veterinarians, and 15 poultry experts from a variety of fields (e.g., animal feed production, academics, and breeding companies) were recruited.

### Questionnaire and Interview Development

The survey consisted of a questionnaire, followed by an interview. The online questionnaire was designed with Microsoft Forms (forms.office.com) and consisted of several closed questions, open questions, and questions with a Likert scale. The questionnaire was developed based on scientific literature on animal welfare assessment (Louton et al., 2017), precision farming (Van Emous, 2021), and disease prevention (Laanen et al., 2014). Questions identified several characteristics of our stakeholders.

Veterinarians and experts filled in 11 questions on sociodemographic information, field of expertise, years of relevant works experience, and frequency of contact with the commercial poultry sector. They additionally answered 5 Likert-scale questions with 6 levels. Levels ranged from completely disagree to completely agree. Likert-scale questions assessed the use of odor, sound, activity and movement, appearance and physical characteristics, and production indicators in the detection of deviations in layer health and welfare.

Farmers filled in 28 questions on their sociodemographic information, years of relevant works experience, details on housing systems, general management characteristics, and the current frequency of logging sensor-based performance and climate data. Farmers answered the same 5 Likert-scale questions as presented to the veterinarians and experts. To determine the indirect need for sensor technology, we developed 3 additional Likert-scale questions with 6 levels. The questions assessed the self-perceived ability of farmers to detect deviations in the health and welfare of the laying hens at an early stage.

In addition to the questionnaire, semistructured interviews were composed based on examples of interviews in the literature in the field of animal welfare and precision livestock farming (e.g., Vaarst, 2003). One laying hen farmer participated in a pilot interview to test and optimize the final interview questions. Interviews were held in December 2021 and January 2022 and lasted on average 30 min with a range of 12–65 min. The narrative interviewing techniques allowed participants to express their opinion freely and to describe their personal experience on all topics (Vigors and Lawrence, 2019). The interview protocol was divided into 4 central objectives, each containing 1 to 3 narrative questions. The 4 objectives are described in the result section. For every narrative question, follow-up probing questions were formulated to inspire involved stakeholders and to raise consistency among participants by touching upon a minimal number of relevant topics. The questionnaire and interview guide were prepared in both English and Dutch.

The interviews were carried out online through Microsoft Teams or in-person. Interviews were voice-recorded. The records were transcribed in full and translated into English when applicable. The transcripts were entered in Atlas.ti (version 22), a software application allowing for qualitative data analysis (de Olde et al., 2020).

### Data Analysis

Answers to open questions in the questionnaire were categorized. The resulting categorial variables were visualized as frequencies. Likert-scale data were treated at an interval level. The answer “completely disagree” received a score of 1, while “completely agree” received a score of 6. Two participants were excluded from the analysis due to missing answers. The mean score and standard deviation were calculated across all stakeholders for the first 5 Likert questions. The mean score and standard deviation were calculated for the remaining 3 Likert-scale questions, which were targeted specifically at laying hen farmers.

The content of the interviews was analyzed using an inductive coding approach as described by de Olde et al. (2020). A coding framework is developed during the analysis, in which codes represent relevant variables deducted from the narrative. A variable-oriented strategy was applied to allow for cross-case analysis (Miles et al., 2014). Variables were summarized, ranked, and presented as percentages across all stakeholders and per stakeholder group for the 4 objectives.

### Ethical Statement

The study adhered to the Wageningen code of conduct for social science research during the conduct of the interview and processing of the data. Participants received a consent form with the description and aim of the project, a justification for data collection, a protocol to store, use and exchange data, and a statement that personal data were anonymized and pseudonymized during data analysis. Participation in the research was on voluntary basis and respondents were able to withdraw during any moment of the study. Voice-records of the interviews were deleted immediately after the transcription was finished.

## RESULTS

In total, 15 poultry experts, 20 laying hen farmers and 9 poultry veterinarians participated in the interview. Of the 44 questionnaires sent out to the participants, 41 were completely filled in and returned (15 poultry experts, 17 laying hen farmers, and 9 poultry veterinarians). Nevertheless, the answers of all 44 participants were incorporated into the result sections belonging to the interview.

The questionnaire results and interview responses are presented below. Detailed results of the questionnaires and interviews, including descriptive tables, can be found in the Supplementary Tables.

### Demographics of Stakeholders

All interviewed farmers were male, while 4 poultry experts and 1 veterinarian were female (Table S1). Stakeholders were found across a wide range of ages (25–65 yr). The majority of stakeholders (68%) had more than 12 yr of work experience. Experts were mainly active in industry (animal feed, genetics, climate, light provision, pharmaceutics), academia, and consultancy. Four experts had a veterinary background (Table S2). Thirteen experts were specialized in (among others) laying hens. Six of the interviewed veterinarians were practitioners, while 3 were nonpractitioners.

### Background Information on Farmers

***Housing.*** Forty-one percent of farmers gave their hens access to an outdoor area with cover. Eighty-two percent of farmers gave their hens access to an outdoor area without cover and thus were considered to be farmers with free-range aviaries (Table S3). Forty-seven percent of farmers had an organic farming system. Flock sizes (per house) varied between 3,000 and 99,000 hens. Eighteen percent of the farmers had an established future successor and 65% were anticipating a potential successor, while 12% knew that their farm would be closed after their departure.

***Management.*** On 65% of the farms, the houses were entered at least twice per day by the farmer, while on the remaining farms, the houses were entered once per day (Table S4). The veterinarian performed a routine visit 1 time or more per month on 18% of farms.

***Registration.*** Feed and water intake were registered daily on all farms; 76% of farmers indicated that they used digital registration, of which 10 farmers additionally wrote the feed and water intake on paper (Table S5). Egg production percentage was registered daily on 53% of the farms, and weekly on the rest of the farms. Seventy-one percent of farmers entered egg production percentage in their digital registration system. Egg weight was registered on 94% of farms, with a frequency of once daily at 24% of the farms. Fifty-nine percent of the farmers registered egg weight digitally. Daily hen weights were registered on 41% of the farms, while 24% of farmers indicated that they did not record hen weight. Three out of 17 farmers only registered hen weight to correctly dose poultry red mite (**PRM**) treatment or specifically at the start of a new flock to follow their first weeks of development. The majority (82%) of farms registered climate parameters, such as temperature and relative humidity, on a daily basis, whereas the other 18% of the farmers did not register climate. Digital registration seemed to be slightly preferred over registration on paper (41 and 29% of the farms, respectively), while 12% used both methods.

### Background Information on Poultry Experts and Poultry Veterinarians

The majority (87%) of the involved poultry experts indicated to visit poultry farms less than weekly (Table S6). Seventy-eight percent of veterinarians indicated to be present on a poultry farm more than once per week, although 56% of these veterinarians mentioned that less than 20% of these visits were to laying hens in aviary systems. Eighty-nine percent of the veterinarians had contact with poultry farmers more than once per week besides physical visits to farms. The majority of the poultry experts and veterinarians reported no decrease in frequency of farm visits and contact with poultry farmers due to Covid-19 or Avian Influenza (**AI**).

### Identification and Prioritization of Indicators of Hen Health and Welfare in the Aviary System

During the interview and preceding questionnaire, stakeholders indicated what they perceived as the most important indicators of optimal and suboptimal health and welfare in laying hens. Indicators could be sorted into 9 different clusters (Table 1). The majority of stakeholders mentioned production parameters (93%), sound (89%), and the behavior of the animals (86%) as indicators for health and welfare assessment in laying hens. In general, deviations in feed intake (61%) and water intake (57%) received most attention. Veterinarians placed additional emphasis on mortality (67%), egg quality (78%), and egg production numbers (67%). The specification of behavioral observations was often missing. Nevertheless, hen activity level (34%), location (distribution and position in the multitier aviary system; 30%), and movement (through the house and aviary system; 30%) were predominantly used as health and welfare indicators. Regarding auditory observations, stakeholders primarily indicated that both too much sound or screams in the house and too little sound or timid sound are signs of suboptimal health and welfare. Veterinarians (44%) additionally listened to abnormal sounds as indication of (respiratory) diseases, such as coughing, sneezing, and gurgling.

**Table 1:** Indicators to determine (suboptimal) health and welfare of laying hen (flocks), identified through the interviews with poultry experts (*n* = 15), laying hen farmers (*n* = 20), and poultry veterinarians (*n* = 9), divided over 9 identified clusters.

Cluster	Indicator description
Production 93% of experts 95% of farmers 89% of veterinarians (93% of all participants) Early warning signal: 80% of all participants Blindspot of farmer: 9% of experts/veterinarians	1. Feed intake
	2. Water intake
	3. Mortality
	4. Egg quality (size, egg shell color/damage/cleanliness/blood spots)
	5. Body weight
	6. Egg production (number of eggs produced)
	7. Egg weight
	8. Floor eggs
	9. Egg production percentage
	10. Uniformity
	11. Feed conversion
	12. Water/feed ratio
	13. Results and remarks as observed in slaughterhouse

Sound 80% of experts 95% of farmers 89% of veterinarians (89% of all participants) Early warning signal: 39% of all participants Blindspot of farmer: 0% of experts/veterinarians	1. Decreased sound level or timid flock
	2. Increased sound level, or screams/whining of chickens
	3. Abnormal sounds, such as coughing, sneezing, wheezing, gurgling, snorting, hiccups
	4. Sound related to stress, aggression and frustration
	5. Sound related to a specific activity (dustbathing, scratching) or heard at a specific time of the day
	6. Sound made by victims of feather pecking

Behavior 93% of experts 80% of farmers 89% of veterinarians (86% of all participants) Early warning signal: 45% of all participants Blindspot of farmer: 7% of experts/veterinarians	1. Activity
	2. Location in the house (distribution on the ground and in the system)
	3. Movement through the house and in the aviary system
	4. Reaction to humans
	5. Attitude (alertness, scared/fearful, stressed, wild, nervous, calm/restless, apathetic)
	6. Feather pecking and feather eating
	7. Mobility/ease of movement
	8. Use of outdoor area
	9. Interaction with conspecifics
	10. Feeding behavior
	11. Drinking behavior
	12. Use of enrichment, such as lucerne bales
	13. Foraging and scratching behavior
	14. Fleeing behavior
	15. Nest use

Appearance Counts: 73% of experts 65% of farmers 89% of veterinarians (73% of all participants)Early warning signal: 11% of all participantsBlindspot of farmer: 13% of experts/veterinarians	1. Feather characteristics (e.g., smoothness, symmetry of feathers, cleanliness, and shine)
	2. Comb characteristics (e.g., size, color, spots)
	3. Feather cover ((localized) baldness)
	4. Posture (e.g., hunched on perch)
	5. Leg and feet health
	6. Eyes (exudate, open/closed)
	7. Toes
	8. Wounds on body
	9. Mouth/beak (e.g., spots in mouth, color of beak)
	10. Vitality
	11. (swollen) Heads

Litter and manure 60% of experts 45% of farmers 78% of veterinarians (57% of all participants) Early warning signal: 14% of all participants Blindspot of farmer: 7% of experts/veterinarians	1. Manure quality (wetness, color, blood, parasites, consistency, undigested feed particles)
	2. Litter quality (wetness, thickness litter, etc.)
	3. Presence or absence of feathers in litter in relationship to laying hen age

Odor 35% of experts 45% of farmers 67% of veterinarians (53% of all participants) Early warning signal: 0% of all participants Blindspot of farmer: 0% of experts/veterinarians	1. Odor as indicator of climate (e.g., ammonia)
	2. Manure quality
	3. Diseases, such as *E. coli*
	4. Dead chickens

Climate and equipment 47% of experts 40% of farmers 33% of veterinarians (41% of all participants) Early warning signal: 2% of all participants Blindspot of farmer: 14% of experts/veterinarians	1. Climate (general)
	2. Checking equipment functionality (e.g., waterline, feedline, egg belt, lights)
	3. Physically feeling the climate (ventilation, temperature, humidity)
	4. Farm facilities (hygiene sluice, egg room, etc.) 5. Outdoor area
	6. Blood on the system, primarily on perches

Physical characteristics 67% of experts 24% of farmers 22% of veterinarians (36% of all participants) Early warning signal: 0% of all participants Blindspot of farmer: 9% of experts/veterinarians	1. General condition (body fat reserves, etc.)
	2. Wing tension
	3. Keel bone health
	4. Crop fill
	5. Laying bones
	6. Comb temperature
	7. Leg asymmetry
	8. Liver quality
	9. Airways
	10. Intestinal health

Other observations and analyses 13% of experts 0% of farmers 45% of veterinarians (14% of all participants) Early warning signal: 9% of all participants Blindspot of farmer: 0% of experts/veterinarians	1. Checking for poultry red mites (in traps, in the system, on the animals themselves)
	2. Checking for worms
	3. Performing dissections (to establish cause of death and to check intestinal health)

Clusters and corresponding indicators are sorted based on highest occurrence. Percentages of respondents reporting indicators from each cluster are provided. Early warning signal means the number of times at which an indicator was marked as earliest/most important warning for suboptimal laying hen health and welfare across all stakeholders (summed per cluster; *n* = 44). Blindspot means blind spots of farmers during flock assessment, as indicated by experts and veterinarians (summed per cluster; *n* = 24).

Seventy-three percent of stakeholders used visual observations regarding the appearance of the hens. Although appearance was not always defined, comb color and size (33%), feather characteristics (30%), and feather cover (11%) received the highest priority. Sixty-seven percent of the experts considered physical characteristics of the hens, such as their general condition and wing tension, as indicators of health and welfare, while only 20% of farmers and 22% of experts mentioned physical characteristics.

Visual indicators of litter and manure quality were mentioned by 57% of the stakeholders. Odor in the house was used by 53% of the stakeholders to assess flock health and welfare status, primarily as indicator for climate conditions (36%). The climate in the poultry house was only mentioned by 20% of the farmers and 33% of the veterinarians. Farmers mostly assessed climate based on physically feeling the ventilation, the temperature and humidity, even if they indicated to register climate digitally.

One of the probing interview questions gained insight into the use of specific warning signals for compromised laying hen health and welfare. For this study, warning signals were defined as “indicators that function as the first and most important sign of deviations on the farm, making the observer (i.e., farmer, expert or veterinarian) worried.” Both productive performance indicators and behavioral observations were considered to be warning signals according to 80% and 45% of all stakeholders, respectively. Specifically, changes in egg size, egg number and egg quality, feed intake, and changes in activity level were marked as early warning signals of compromised health and welfare.

Experts and veterinarians identified 3 categories of blind spots that farmers can have during flock control. The first category was indicators that lack a reference in optimal conditions. Examples were animal-based observations, including feather cover and injuries (Cluster: Appearance), piling, reduced activity and social behavior (Cluster: Behavior), and keel bone damage (Cluster: physical characteristics). The second category was observations that do not trigger necessary actions by the farmer. Climate parameters such as temperature and relative humidity were often recorded, but not analyzed by farmers to steer management decisions. The third category was indicators of which farmers don't know the (correct) relationship to health and welfare, which were mostly physical characteristics.

**Figure 1:**
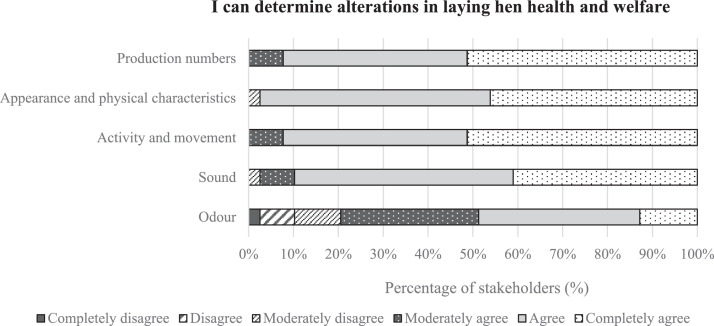
The self-perceived ability of poultry experts (*n* = 13), laying hen farmers (*n* = 17), and poultry veterinarians (*n* = 9) to determine alterations in laying hen health and welfare, based on 5 predefined categories of indicators, and scored on a 6-point Likert scale from completely agree to completely disagree.

Next to the indicators derived from the interviews (Table 1), the online questionnaire also included a question on the ability of farmers to determine alterations in animal health and welfare based on 5 predefined categories of indicators. Predefined categories differed from the categories that evolved from the interviews (Figure 1). Stakeholders had most confidence in determining alterations in health and welfare based on “Production,” “Activity and movement,” and “Appearance and physical characteristics” (all score: 5.4), followed by sound (score: 5.3). In general, the lowest value was given to “Odor” (score: 4.3), with the highest variation across stakeholders (SD = 1.2).

### Identification and Prioritization of Relevant Health and Welfare Issues, Including Their Causal Stressors and Risk Factors in the Aviary System

During the interview, stakeholders indicated what they perceived as the most important health and welfare issues. These issues were observed on the laying hen farmer's own farm, at clients of veterinarians and experts or in the laying hen sector, in general. Issues could be sorted into 7 clusters (Table S7). Table 2 shows the 10 most frequently reported issues for all 3 stakeholder groups, and includes the perceived relevance and associated risk factors of each issue. *Escherichia coli* was most consistently perceived as a relevant health problem (52%). *E. coli* relevance was determined by its high prevalence on laying hen farms. According to stakeholders, an *E. coli* outbreak does not appear every flock cycle and is therefore an intangible problem. *E. coli* was associated with increased mortality and sudden appearance in a flock. The emergence of *E. coli* was associated with suboptimal climate conditions, such as draughts and sudden changes in temperature between night and day. PRM (*Dermanyssus gallinae*) and FP were the second most frequently mentioned problems (both 50%). The effect of PRM on hen health and welfare was thought to be underestimated, in part because of the subclinical nature of the infection. Infection was associated with the transmission of other diseases and the absence of effective treatments.

**Table 2:** Overview of the indicated most relevant health and welfare problems in the laying hen sector, based on selecting the 5 most frequently mentioned issues for 3 stakeholder groups (poultry experts, laying hen farmers, and poultry veterinarians).

Health and welfare issue	Reasons determining the relevance	Associated risk factors
*Escherichia coli* 33% of experts 55% of farmers 78% of veterinarians 52% overall	Prevalent Strong association with increased mortality Suddenly appearing/ fast spread among flocks Variable occurrence across farms/ flocks	(Changes in) climate (seasonal/between day and night) Ventilation
*Dermanyssus gallinae* 60% of experts 40% of farmers 56% of veterinarians 50% overall	Transmission of diseases Noneffective/expensive/nonexistent treatment Overlooked/subclinical Underestimated	
Feather pecking 73% of experts 40% of farmers 33% of veterinarians 50% overall	Prevalent Multifactorial Subjected to public opinion	Untreated beaks Feed quality and composition Suboptimal intestinal health Restriction outdoor access
Intestinal disorders 27% of experts 45% of farmers 56% of veterinarians 41% overall	Reoccurrence related to switch from cages to free-range housing systems Overlooked/subclinical	Feed quality and composition
Infectious bronchitis 20% of experts 30% of farmers 67% of veterinarians 34% overall	Prevalent High impact	Multiage farm Outdoor access Poultry-dense area
Worms 20% of experts 45% of farmers 33% of veterinarians 34% overall	Noneffective/expensive/nonexistent treatment	Alternative housing Outdoor access Walking in manure
Climate 33% of experts 25% of farmers 56% of veterinarians 34% overall	Prevalent High impact	Outdoor access
Avian influenza 33% of experts 25% of farmers 0% of veterinarians 23% overall	Current importance High impact Complex/multifactorial/nonexistent prevention Suddenly appearing/ fast spread among flock	Outdoor access Poultry-dense area
Cannibalism 33% of experts 10% of farmers 33% of veterinarians 23% overall	Increasing in prevalence over the years	Outdoor access White layer breeds
Bone-related issues 40% of experts 5% of farmers 22% of veterinarians 20% overall	Variable occurrence across farms/ flocks	Feed quality and composition Alternative housing system High-productive laying hens

Percentages of stakeholders reporting each issue are provided. The overview includes the reasons given by stakeholders to mark the issue as relevant, and the perceived risk factors.

Intestinal disorders, worm infections, climate problems, and infectious bronchitis received intermediate attention across stakeholders (between 25 and 50%). Less than 25% of stakeholders mentioned AI, cannibalism and bone-related issues as important health and welfare issues.

Interestingly, veterinarians did not mention AI as a relevant issue. Only a few (27%) experts mentioned intestinal disorders, compared to 45% of farmers and 56% of veterinarians.

### Assessment of Current Sensor Technology Use in Commercial Production

Laying hen farmers were asked about currently collected (sensor) data on their farm. Most farmers tracked feed intake (95%) and water intake (90%) and the climate (i.e., temperature and relative humidity; 85%), followed by automatic collection of egg characteristics (65%) and animal weight (35%) (Table 3). Fifteen percent of farmers acquired management software, namely Porphyrio, Farmconnect, Myfarm, and Farmresult. Digital or paper records on mortality and PRM infestation were considered as sources of hen health and welfare data. Other technologies included those that control the climate and egg collection belt based on actual measurement data. Farmers mainly used (sensor-based) collected data to facilitate and optimize daily management decisions, to visualize the development of production parameters over time, and to compare the productivity of different rounds of flocks.

**Table 3:** Data and techniques reported by laying hen farmers in response to the question: “Which (sensor) data are currently collected on your farm?”. Responses are divided into 3 themes (sensors, tools and records, and other technologies).

Sensors	Reported	Specification
Climate sensors	85%	CO_2_, relative humidity, temperature, negative pressure
Information on eggs	65% (10%)^1^	Provided by egg buyer, own egg counters, and/or Meggsius and/or the use of electronic eggs. Information includes egg number, egg weight, egg quality (e.g., breaking strength, egg size, etc.)
Animal weight	35% (10%)^1^	
Feed intake	95%	
Water intake	90%	
Cameras	5%	In the barn for checking the laying hens and their distribution
Fire detector	5%	
Management software	15%	For example, Porphyrio, Farmconnect, Myfarm, Farmresult
Paper performance card	15%	
Record of mortality	40%^1^	
Poultry red mite counts	5%^1^	
Other technologies		Specification
Machinery to control the climate	30%	Ventilation systems, ECO Unit, etc.
Technology in egg belt	5%	For example, to start and stop the system and adjust the speed of the egg belt

1 Data that are acquired or registered manually only.

### Identification of Advantages, Wishes, and Obstacles Regarding the Future Use of Sensor Technology for Daily Management

**Figure 2:**
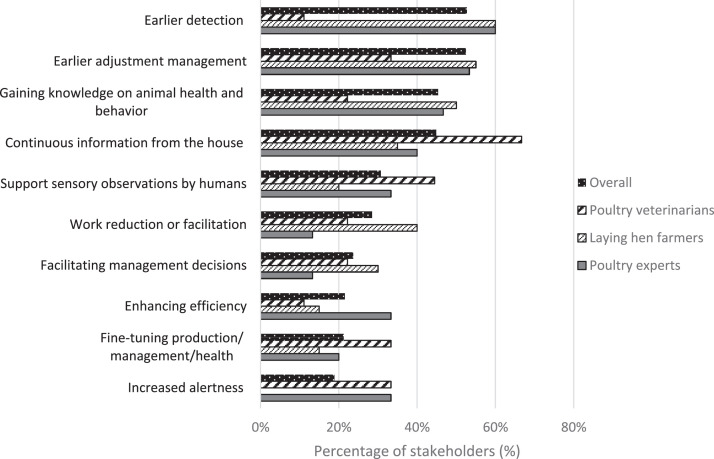
Ten most frequently reported advantages of sensor technology and automation according to poultry veterinarians (*n* = 9), laying hen farmers (*n* = 17), and poultry experts (*n* = 15) and across all stakeholders (*n* = 44).

***Advantages.*** The 2 most important indicated advantages of sensor use in the laying hen house were earlier detection of deviations in the house (53%) and earlier adjustment of the management based on sensor data (53%; Figure 2). Farmers (50%) and experts (47%) additionally emphasized the potential of sensors to acquire new knowledge on animal health and welfare. Veterinarians emphasized having continuous insight into the flock status (67%) and supporting sensory observations that are now performed by humans (44%). Experts and veterinarians envisioned that sensors could increase the alertness of both farmers and themselves when walking through the barn (33% each), but increased alertness was not mentioned as benefit by laying hen farmers.

***Needs and Wishes.*** Needs and wishes for sensor technology were inferred from the stakeholders’ narratives during the interview. Four predominant needs for sensor technology could be derived. First, several tasks on the farm were considered as demanding in terms of work load, time, and work pleasure. Examples include the logging and collection of data on the computer, the collection of eggs outside the nests and the collection of dead animal in the nests. Second, stakeholders mentioned that specific data or information is missing (i.e., currently not collected), especially regarding hen behavior around egg laying, egg quality, continuous animal weights, ammonia, and CO_2_. Third, reductions in staff availability and expertise stressed the need for sensor technology developments, especially in large-scale operations outside Europe, but also in smaller scale farms in the Netherlands. Fourth, there was a need for reduced subjectivity in health and welfare assessment.

To respond to these inferred needs, experts, and veterinarians envisioned a monitoring system in which poultry farmers receive clear guidance and advice after detection of (major) deviations in the laying hen house, to avoid ignoring or worrying about the detected deviation. A warning mechanism should be part of the system, to encourage people to pay extra attention and to perform additional checks in the house. According to expert 14; *“Proactively looking at data is already a problem, and it will be a problem in the future if no signals are generated.”* Almost half of all experts wished for a system that made autonomous adjustments in the house, although they recognized that these are long-term wishes. However, veterinarians and farmers did not envision autonomously reacting systems. Experts largely focused on integration of different data sources and linking the climate computer to other systems, such as the animal weighers or the egg packers. Experts saw potential in creating one comprehensive overview with all data, including (changes in) management decisions. The overview should allow farmers to easily look back into the flock history. Compared to experts and veterinarians, farmers put slightly more focus on an alarming system than on the advisory role of the system. Farmer 7*: “A warning system first, because you visit the house every day anyway. Then you can look at what the system indicated and what you see yourself.”* All stakeholder groups wished for clear visualization of data, including trendlines, graphs, and figures, in which small deviations are visible.

**Figure 3:**
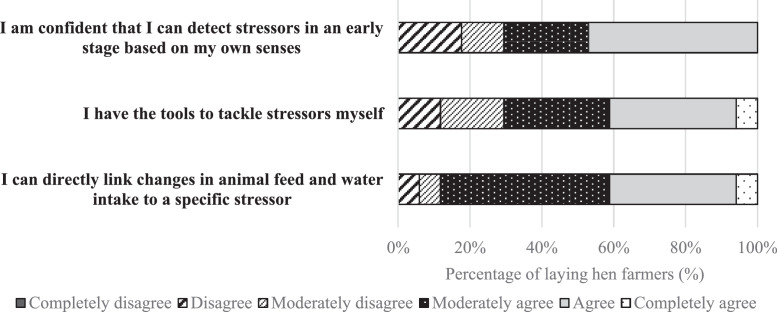
The perception of laying hen farmers (*n* = 17) on their ability to detect, identify, and tackle stressors in laying hens, score on a 6-point Likert scale from completely agree to completely disagree.

In the questionnaire, 3 Likert-scale questions were proposed to laying hen farmers to identify the indirect need for sensor technology based on their self-perceived ability to detect, identify, and tackle stressors. Twenty-nine percent of farmers did not agree that they could detect stressors at an early stage based on own observations in the laying hen house (Figure 3). Twenty-nine percent of farmers reported not to have the tools to tackle stressors themselves. Feed and water intake provided an indication of stress according to 88% of the farmers.

### Obstacles

**Figure 4:**
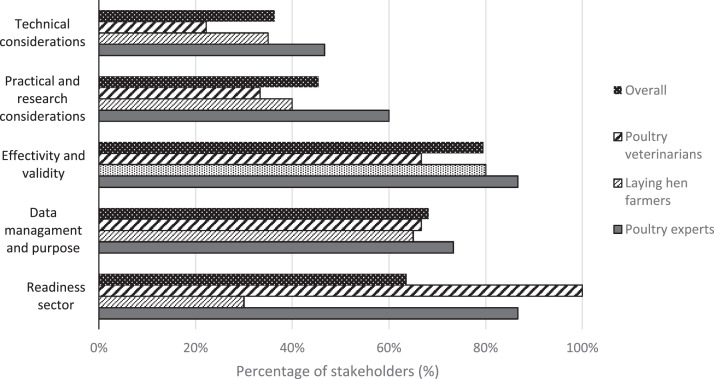
Percentage of poultry veterinarians (*n* = 9), laying hen farmers (*n* = 20), poultry experts (*n* = 15), and all stakeholders (*n* = 44) reporting on 5 potential obstacles for successful implementation of sensor technologies in the laying hen sector.

Stakeholders saw the effectivity and validity of sensor-based health and welfare assessment as the largest obstacle (80%) (Figure 4). The uncertainty about sensor effectivity and validity arose from the doubt whether or not financial investments would be outweighed by the benefits of sensors in practice. Veterinarians explicitly expressed their concerns regarding the readiness of the sector for sensor technologies (100%), caused by among others the perceived limitations of sensors to replace human observers (Table S8). Interestingly, few farmers (30%) reported obstacles regarding readiness of the sector. Several farmers (35%) were afraid that the government and other organizations would use data for the wrong purpose or misinterpret data. Experts seemed unsure whether or not farmers would make use of all technology and dashboard functions.

### Suggested Sensors and Sensor Research

**Figure 5:**
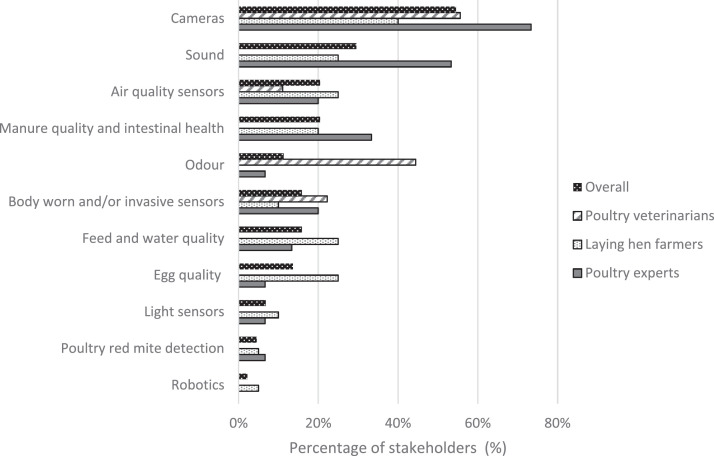
Percentage of poultry veterinarians (*n* = 9), laying hen farmers (*n* = 17), poultry experts (*n* = 15), and all stakeholders (*n* = 44) who reported their interest in 11 identified research fields for future sensor use in the laying hen sector.

Stakeholders expressed their interest in future technologies and in future research into several parameters of interest, such as manure quality and water quality (Table S9). Stakeholders had most interest in the use of cameras (55% of participants) and microphones (30%) for continuous monitoring of laying hen health and welfare (Figure 5). Specifically, stakeholders expressed their interest in the use of (thermographic) cameras for observation of specific behaviors, the distribution of birds and for monitoring bird activity level. Microphones were thought to be useful to measure loudness in the barn, pecking sounds, and specific communication calls, such as the gakel call (Zimmerman and Koene, 1998). Other suggested sensors were related to monitoring air quality (20%), primarily ammonia and fine dust concentrations. Moreover, sensors to monitor egg quality (14%) and feed and water quality (16%) were suggested for further development and application. The highest variance between stakeholder groups was seen for the development of odor sensors; none of the farmers suggested the development of an odor sensor, compared to 44% of veterinarians and 7% of poultry experts.

## DISCUSSION

The aim of this study was to capture the current thinking of stakeholders in the poultry sector regarding current and potential future use of sensors on poultry farms. Several selection criteria were adhered to, in particular regarding farmers who had a focus on innovation and showed strong involvement in daily farm management.

### Production, Behavior, and Sound

Production indicators were most commonly used across stakeholders to assess hen health and welfare, with nuanced differences between stakeholder groups that might stem from a differential definition of the concept “welfare”. The majority of laying hen farmers focused on the use of feed intake and water intake and showed high confidence in detecting health and welfare deviations based on these parameters. Livestock farmers consistently perceive welfare as the minimization of health issues (Kauppinen et al., 2010). Positive animal welfare is often linked to adequate resource provision, such as good feed and water (Vigors and Lawrence, 2019). Others suggested that animal welfare is perceived as “an additional area of interest” in addition to productivity, and that assessment of welfare is only important when things go wrong in the barn (Vaarst, 2003).

According to the stakeholders, the interpretation of currently available production data is lacking, and there is more potential in the use of production data in decision-making during daily management. New initiatives in the poultry sector include digital platforms to store management data in one place, and sensors to measure egg size and external quality. According to the veterinarians, egg quality, egg size, and egg production numbers are production indicators that can serve as an early warning for suboptimal health and welfare. Stressors, such as diseases and physical impairments, can cause a reduction in egg production due to energy reallocation (Wang et al., 2017). Sleeckx et al. (2019) showed that infestations with PRM could decrease laying percentage, egg weight and mass and increase the number of second-choice eggs. Morales et al. (2016) stated that production problems could be detected 1 d in advance based on the analysis of egg production curves with machine learning techniques.

While farmers considered egg production and quality a good indicator of health and welfare, farmers differed on the value of natural behaviours in assessing health and welfare. Organic farmers generally assign a higher value to the expression of natural behavior than conventional farmers (Skarstad et al., 2007; Vigors and Lawrence, 2019). In the current study, organic farmers were over-represented at the expense of conventional farmers, which might explain the high use of behavioral indicators in the assessment of hen health and welfare. More specifically, important behavioral indicators were general activity and movement of hens through the aviary, and the response of animals to human approach. Experts and veterinarians mentioned animal-based observations, such as behavior, as blind spots during farm management. Suboptimal use of animal-based observations could justify the development of continuous sensor techniques focused on hen behavior. Individual hen movement patterns (i.e., the number of movements to specific zones of the aviary and in total) are highly consistent in time (Rufener et al., 2018). Future research could explore whether or not changes in rhythmicity patterns of vertical and horizontal movement could be used to detect emerging health and welfare problems in laying hens at an early stage (Stachowicz and Umstätter, 2021).

Farmers, but also veterinarians seemed to prioritize the sound of the hens over for example hen appearance. Stakeholders agreed that high levels of noise and screams, or quiet flocks with timid sounds, are late indicators of suboptimal health and welfare. However, they recognized the potential in the objective analysis of sound for health and welfare assessment, and recurrently suggested the development of microphones to support farm management. Sensor-based sound analysis could fulfill the wish to have reduced subjectivity in daily health and welfare assessment. Sound is a potential indicator for FP (Bright, 2008), viral diseases (Banakar et al., 2016), and thermal comfort (Du et al., 2020). Sound levels in PRM-infested poultry houses are commonly reported to be increased (Sigognault Flochlay et al., 2017), which suggests an opportunity for sound analysis in PRM monitoring.

### Health and Welfare Problems

A recent review identified welfare issues in free range and organic laying hen flocks in Europe based on literature and expert interviews (Bonnefous et al., 2022). The study highlighted the enhanced risk of bacterial, viral and parasitic infection in layer flocks with outdoor access. In our study, *E. coli* received the most attention during the interviews out of all health and welfare issues, especially among farmers and veterinarians. Infection by *E. coli* is a common cause of mortality for layers housed in alternative housing systems (Fossum et al., 2009). Several farmers were aware that suboptimal climate conditions predispose the outbreak of *E. coli* infections. Veterinarians and poultry experts, however, identified climate control as a blind spot of laying hen farmers. Farmers mostly referred to climate control as “feeling the ventilation,” even though their farm was equipped with climate sensors. Adequate training and objective help should be provided to farmers to enable them to run systems and optimally use data to steer management (Hartung et al., 2017). Visualization through comprehensable user interfaces will increase the added value of sensor technologies, especially when information from several sensors are combined in one platform and are linked to early warning signals (Van Hertem et al., 2017).

The high ranking of PRM among stakeholders is mainly due to the absence of effective, affordable treatments, the late diagnosis due to subclinical symptoms and its role as a disease vector. Self-assessed infestation rates on commercial laying hen farms differ from mite trap-based infestation rates (Waap et al., 2019), resulting in underestimation of infestation load. Schreiter et al. (2022) identified PRM load as a risk factor for severe FP. In our study, FP was appointed as the most relevant health and welfare problem in hens housed in aviaries by poultry experts. Poultry experts appointed outbreaks of FP and cannibalism as relevant due to their severity when no swift actions are taken (Rioja-Lang et al., 2020). Although less prominently compared to experts, several farmers saw FP as a prevalent problem, with a multifactorial origin that is highly subjected to the public opinion. FP is thought to be a redirected foraging behavior that is linked to inadequate access to foraging substrates (Rodenburg et al., 2013), and there seems to be a negative relationship between the use of the outdoor run and the occurrence of severe FP (Bestman and Wagenaar, 2003). High ranking of FP among farmers could be linked to the large number of farmers who were obliged to restrict outdoor access to their hens due to the emergence of AI at the time of the interview. Farmers with brown hens tended to report more on FP than farmers with white hens, which is in line with evidence for higher incidence of FP in brown vs. white hen breeds (Kjaer, 2000).

Interestingly, veterinarians and experts considered FP to be a blind spot of farmers. They argued that laying hen farmers underestimate the value of poor feather cover as indicator of decreased health and welfare, since it might be considered as a normal phenomenon related to aging of the hens (Kaukonen and Valros, 2019). Farmers’ flock size seemed to negatively relate with the use of appearance indicators, although this relationship was based on a limited number of observations. This relationship could nevertheless suggest that assessment of hen appearance demands a higher time investment than the insight it gives into the flock status (Kaukonen and Valros, 2019).

Less than half of the stakeholders considered intestinal disorders as relevant in the laying hen sector. Intestinal disorders can remain subclinical or cause general symptoms like reduced egg production (Roberts et al., 2011). Clinical symptoms of intestinal disorders include abnormal droppings with high moisture levels. Wet droppings can form a threat to food safety by soiling the egg shell. Few stakeholders mentioned the ability to use odor in the house as an indicator of intestinal disorders. Previous studies have shown the potential of odor monitoring for the detection of Salmonella-infected broilers (Kizil et al., 2015) and coccidiosis (Borgonovo et al., 2020). Future studies could provide detailed evidence for the relationship between hen health and welfare, and odor in poultry houses.

Surprisingly, bone-related issues, such as keel bone fractures, did receive attention by less than 25% of the stakeholders. Prevalence rates of keel bone fractures on commercial laying hen farms have been reported as high as 97% in Belgium and 86% in the Netherlands (Rodenburg et al., 2008). In current practice, the assessment of keel bone fractures requires manual handling of hens. Future monitoring techniques could predict keel bone fractures based on detecting decreased numbers of hen transitions between aviary zones, since keel bone fractures can reduce a hen's mobility (Armstrong et al., 2020).

### Sensor Opportunities

The 2 most important advantages of sensor (data) use in the laying hen house were earlier detection of deviations in health and welfare indicators and consequently, earlier adjustment of the management. Although some farmers used management software to store flock history, they did not feel that the current design allows for data-driven decision making as the measurements do not have a predictive value yet. The current collection and use of data seemed to vary between farmers; several farmers did not look at digital records of egg production percentage, climate, and egg weights. The majority of respondents were, however, aware of the potential of the collected data when integrated into one platform and visualized into clear graphs and figures.

Several farmers envisioned the development of a warning system to detect decreased health and welfare. However, their answers suggested that their vision of technology is mostly focused on automation to assist during time-demanding tasks, such as entering data in the computer and collection of floor eggs. The prioritized wishes could resonate with recent developments in the Netherlands, including the lack of personnel with a background in poultry management and the outbreak of highly pathogenic AI on poultry farms. Veterinarians and experts alternatively favored a detection system that provided farmers with advice and guidance to ensure that actions are taken when problems are detected. As suggested by Niloofar et al. (2021), data-driven support systems should not only be based on real-time data, but also the expertise of experts to allow for optimal decision making with reduced risk on poor decisions. Despite the proposed advantages of sensor technologies for animal welfare, PLF technologies still have to prove themselves in practice and it is important to be aware of potential direct and indirect threats of sensor technologies for the animals (Tuyttens et al., 2022). Our respondents were most aware of the direct dangers of sensor technology to the laying hens in case of technical failures, inaccurate predictions and decisions due to limited external validity. Identified indirect threats were that farmers might spend less time around the animals, lose their husbandry skills and become over-reliant on PLF technology. de Olde et al. (2020) presented several obstacles for the development and implementation of innovations in the Dutch egg sector, based on interviews with Dutch stakeholders. One of the main obstacles was financial resources and higher labor investment, which was also brought forward in the current study. Lack of farm succession was brought forward as a limitation for the implementation of innovations (de Olde et al., 2020). In our study, the presence or absence of a successor did not seem to have impact on current or future perspectives on monitoring techniques. Conversely, the farming system seemed to affect the number of obstacles that were brought forward; organic farmers mentioned more obstacles than conventional farmers, but they also gave more suggestions for future sensor technology. Together, these findings suggest that organic farmers are more familiar with or more interested in the concept of sensors and precision livestock farming than conventional farmers. Farmers recognized the added value of early on-farm detection tools to allow for fast adjustment of the management to minimize the impact of stress on the hens. Currently, farmers and poultry experts missed fundamental behavioral information of the laying hen flock, and new sensor technologies might be used to gain new knowledge on, for example, nesting behavior to better understand the phenomenon of floor eggs.

In conclusion, this study identified the perception of stakeholders on laying hen health and welfare indicators, including the prioritization and the context of these indicators. Current health and welfare assessment is primarily based on daily inspection of production parameters, listening to the sound when entering the poultry house, and observation of hen activity and distribution in the house. Current sensor(data) use is low on commercial aviary farms.

Individual hen parameters, such as egg quality parameters, can be integrated with continuous flock-based parameters, such as activity and sound monitoring. The added value of these technologies for early detection of relevant health and welfare problems can be increased by incorporating different stakeholder perspectives into sensor development, thus enhancing sensor acceptability. The short-term value of sensors mainly lies in automation to assist during time-demanding tasks, which lowers the barrier to implement these products in practice. Ultimately, sensors can prove their potential in early detection of decreased hen health and welfare in the long-term.

## Appendix Supplementary materials

Image, application 1

## Footnotes

Supplementary material associated with this article can be found in the online version at doi:10.1016/j.psj.2023.102581.
