# Supplementary material for: Perception of laying hen farmers, poultry veterinarians, and poultry experts regarding sensor-based continuous monitoring of laying hen health and welfare
Source: Poult Sci. 2023 Feb 13;102(5):102581. doi: 10.1016/j.psj.2023.102581 (PMC10027566; doi:10.1016/j.psj.2023.102581)
Supplement: Supplementary file 1 [file mmc1.docx]

SUPPLEMENTARY DATA

**Table S1.** Sociodemographic information of poultry experts, laying hen farmers and poultry veterinarians. Country of work and gender are presented for all stakeholders (n=44), while the other information is presented only for the stakeholders that participated in the online questionnaire (n=41). Results are presented as percentages

|  | **Poultry experts** | **Laying hen farmers** | **Poultry veterinarians** | **Total** |
| --- | --- | --- | --- | --- |
| **Country of work** | n=15 | n=20 | n=9 | n=44 |
| **Netherlands** | 93% | 85% | 89% | 88% |
| **Belgium** | 7% | 5% | 0% | 5% |
| **Germany** | 0% | 10% | 0% | 5% |
| **Canada** | 0% | 0% | 11% | 2% |
| **Gender** |  |  |  |  |
| Female | 27% | 0% | 11% | 12% |
| Male | 73% | 100% | 89% | 88% |
|  | n=15 | n=17 | n=9 | n=41 |
| **Age (years)** |  |  |  |  |
| ≤ 24 | 0% | 6% | 0% | 2% |
| 25-34 | 20% | 18% | 22% | 20% |
| 35-44 | 13% | 18% | 11% | 15% |
| 45-54 | 20% | 24% | 33% | 24% |
| 55-64 | 47% | 35% | 11% | 34% |
| ≥ 65 | 0% | 0% | 22% | 5% |
| **Relevant work experience (years)** |  |  |  |  |
| None | 7% | 0% | 0% | 2% |
| <3 | 7% | 0% | 0% | 2% |
| 3 to 6 | 7% | 18% | 11% | 12% |
| 6 to 12 | 20% | 6% | 22% | 15% |
| 12 to 24 | 20% | 29% | 22% | 24% |
| >24 | 40% | 47% | 44% | 44% |

**Table S2.** Professional background of poultry experts and poultry veterinarians

|  | **Poultry experts** (n=15) |  | **Poultry veterinarians (n=9)** |
| --- | --- | --- | --- |
| **Current profession** |  | **Current profession** |  |
| Poultry specialist | 12 | Consulting poultry veterinarian | 1 |
| Other | 3 | Practicing poultry veterinarian | 6 |
|  |  | Other | 2 |
| **Specialization** |  | **Specialization** |  |
| Only layers | 7 | Only layers | 3 |
| Only broilers | 1 | Only broilers | 2 |
| Both | 6 | Both | 4 |
| Other/none | 1 | Other/none | 0 |
| **Veterinary background** |  |  |  |
| yes | 4 |  |  |
| no | 11 |  |  |

**Table S3.** Descriptive characteristics of housing variables of laying hen farmers (N=17), collected in the online questionnaire

| **Housing** | **Percentage** | **Housing** | **Percentage** |
| --- | --- | --- | --- |
| **Housing system** |  | **Number of houses per farmer** |  |
| Aviary | 100% | 1 | 47% |
| Outdoor access with cover | 41% | 2 | 24% |
| Outdoor access without cover | 82% | 3 | 18% |
| **Hen type** |  | 4 | 6% |
| White | 71% | 5 | 6% |
| Brown | 76% | **Feeding system** |  |
| of which both at same time | 41% | Chain feeding | 88% |
| **Economic farming system** |  | Spiral feeding | 29% |
| Conventional | 76% | Both systems in different houses | 12% |
| Organic | 47% | **Water system** |  |
| **Number of hens/house** |  | Nipples | 100% |
| ≥3,000 to < 15,001 | 41% | Cups | 18% |
| ≥15,000 to < 27,000 | 53% | Both systems in different houses | 18% |
| ≥27,000 to <39,000 | 35% | **Successor** |  |
| ≥39,000 to <51,000 | 24% | Yes | 18% |
| ≥51,000 to <63,000 | 18% | No, my farm will be closed | 12% |
| ≥63,000 to <75,000 | 6% | No, maybe in the future | 65% |
| ≥75,000 to <87,000 | 12% | No, I am the successor | 6% |
| ≥87,000 to <99,000 | 6% |  |  |

**Table S4.** Descriptive characteristics of management variables of laying hen farmers (N=17), collected in the online questionnaire

| **Management** | **Percentage** |
| --- | --- |
| **Feeding frequency at the start of peak production** |  |
| ≤ 5 times per day | 71% |
| ≥ 6 times per day | 29% |
| **Use of enrichment** |  |
| Lucerne | 88% |
| Pecking stones | 94% |
| Other (grain scattering, feed scattering, straw provision | 47% |
| **Frequency of entering house** |  |
| Daily | 35% |
| ≥ 2 times daily | 65% |
| **Routine visit by veterinarian** |  |
| < 1 times per month | 82% |
| 1 time per month | 12% |
| > 1 time per month | 6% |

**Table S5.** Descriptive characteristics of variables regarding registration of farm data by laying hen farmers (N=17), collected in the online questionnaire

| **Registration** | **Percentage** |
| --- | --- |
| **Feed/water** |  |
| Frequency: daily | 100% |
| Method: Paper | 82% |
| Method: Digital | 76% |
| Method: both paper and digital | 59% |
| **Egg production percentage** |  |
| Frequency: daily | 53% |
| Frequency: weekly | 47% |
| Method: Paper | 76% |
| Method: Digital | 71% |
| Method: both paper and digital | 47% |
| **Egg weight** |  |
| Frequency: daily | 24% |
| Frequency: weekly | 71% |
| I do not register egg weight | 6% |
| Method: Paper | 71% |
| Method: Digital | 59% |
| Method: both paper and digital | 35% |
| **Hen weight** |  |
| Frequency: daily | 41% |
| Frequency: weekly | 18% |
| I do not register animal weight | 24% |
| I only register hen weight at certain ages/for adjusting treatments | 18% |
| Method: Paper | 35% |
| Method: Digital | 41% |
| **Climate** |  |
| Frequency: daily | 82% |
| I do not register climate | 18% |
| Method: Paper | 41% |
| Method: Digital | 53% |
| Method: both paper and digital | 12% |

**Table S6.** Frequency of contact of poultry experts and poultry veterinarians with poultry and poultry farmers

| **Contact poultry (farmers)** | **Poultry experts (n=15)** | **Poultry veterinarians (n=19)** |
| --- | --- | --- |
| **Frequency poultry farm visit (all poultry species)** |  |  |
| More than once per week | 13% | 78% |
| More than once per month | 40% | 22% |
| Less than once per month | 47% | 0% |
| **of which to laying hens in aviaries** |  |  |
| No visits to aviaries | 7% | 0% |
| Less than 20% | 33% | 56% |
| Between 49 % - 20 % | 13% | 0% |
| Between 79 % - 50 % | 13% | 33% |
| ≥ 80% | 33% | 11% |
| **Contact poultry farmers (e.g. via telephone, on trade events)** |  |  |
| More than once per week | 40% | 89% |
| More than once per month | 47% | 11% |
| Less than once per month | 13% | 0% |
| **Change farm visit** |  |  |
| No change | 53% | 78% |
| Reduction due to Covid-19 | 27% | 11% |
| Reduction due to AI | 13% | 0% |
| **Change contact poultry farmers** |  |  |
| No change | 93% | 89% |
| Increase | 7% | 11% |

**Table S7.** Health and welfare issues identified by poultry experts, laying hen farmers and poultry veterinarians. Absolute counts are presented, specified per stakeholder group and across all stakeholders. Issues are sorted into 7 categories

| **Health and welfare issue** | **Poultry experts (n=15)** | **Laying hen farmers (n=20)** | **Poultry veterinarians (n=9)** | **Total (n=44)** |
| --- | --- | --- | --- | --- |
| **Pathogens** | | | | |
| Bacterial and viral diseases  AI  IB  Coryza  Clostridium  Pasteurella  *Escherichia coli*  Turkey Rhinotracheitis  Mycoplasma  Erysipelas  Salmonella | 2  5  3  0  1  0  5  0  0  1  1 | 2  5  6  1  3  0  11  0  1  0  1 | 0  0  6  2  3  4  7  1  2  3  2 | 4  10  15  3  7  4  23  1  3  4  4 |
| **Pests (internal and external)** | | | | |
| Pests  Coccidiosis  Poultry red mite  Mycotoxins  Worms | 0  1  9  0  3 | 2  1  8  1  9 | 0  4  5  1  3 | 2  6  22  2  15 |
| **Physical and physiological** | | | | |
| Digestive issues  Intestinal disorders  Respiratory issues  Bone issues  Leg and feet issues  False layer syndrome  Heat stress  Nutrient deficiency  Post-peak dip  Egg shell quality  Feed and water intake  Fatty liver | 0  4  2  6  2  0  2  0  1  1  0  1 | 5  9  0  1  0  1  0  1  0  0  1  0 | 1  5  1  2  2  0  0  1  0  0  1  0 | 6  18  3  9  4  1  2  2  1  1  1  4 |
| **Behavior** | | | | |
| Boredom  Fleeing behavior  Drumming  Limited use outdoor area  Piling behavior  Toe pecking  Cannibalism  Feather pecking  Other (negative) social interaction | 1  0  2  0  2  2  5  11  2 | 0  1  5  0  0  4  2  8  0 | 0  0  2  1  1  1  3  3  0 | 1  1  9  1  3  7  10  22  2 |
| **Housing and management** |  |  |  |  |
| Climate  Manure belt aeration  Litter (quality)  Water quality  Traces disinfectants  Alternative products  Feed quality | 5  1  0  1  0  0  0 | 5  0  0  0  0  0  0 | 5  1  1  2  1  1  3 | 15  2  1  3  1  1  3 |
| **Economic and labor** |  |  |  |  |
| Financial  Late laying hens  Floor eggs | 1  0  2 | 2  1  5 | 0  0  1 | 3  1  8 |

**Table S8.** Obstacles to implement sensor based monitoring tools in laying hen houses mentioned by poultry experts (n=15), laying hen farmers (n=20) and poultry veterinarians (n=9), divided into five clusters

| **Cluster** | **Obstacles** |
| --- | --- |
| **Readiness sector** |  |
|  | Readiness of the sector to get involved in innovations (n=10) |
|  | Willingness to use (all) sensor functions (n=9) |
|  | Target market (n=7) |
|  | Less positive human- animal interaction (n=3) |
|  | Limited chain integration (n=2) |
|  | Unknown responsibility (n=1) |
| **Data management and purpose** |  |
|  | Data ownership (n=13) |
|  | Data privacy (n=10) |
|  | Misinterpretation and wrong intentions with data (n=14) |
|  | Data exchange (n=8) |
|  | Enhanced control by government (n=4) |
|  | People caught on camera/microphone (AVG) (n=4) |
|  | Opportunity for hackers (n=3) |
|  | Data ownership (n=13) |
| **Effectivity and validity** |  |
|  | Economical obstacles (n=22) |
|  | Limitation of sensors to replace humans (n=11) |
|  | Return for data (n=7) |
|  | Ease of use system and visualization (n=7) |
|  | Extra work (n=5) |
|  | Inadequate guidance and education (n=4) |
| **Practical and research considerations** |  |
|  | Reliability of sensors and false alarms (n=8)  Complicated data analysis, interpretation and validation (n=6) |
|  | Flock comparison and standardization (n=4) |
|  | Research limitations (n=3) |
|  | Competition from other research groups (n=2) |
|  | Lack of fundamental animal knowledge (n=1) |
|  | Invasive for the animals (n=1) |
| **Technical considerations** |  |
|  | Robustness and lifespan (n=7) |
|  | Technical considerations (n=6) |
|  | Communication between systems and ease of data collection (n=5) |
|  | Integration into current system (n=3) |

**Table S9.** Specification of research areas in relationship to sensor based monitoring techniques that were mentioned by poultry experts (n=15), laying hen farmers (n=20) and poultry veterinarians (n=9) during the interviews

| **Focus area of sensor** | **Specification** |
| --- | --- |
| Air | Movement and speed, quality, such as (fine) dust, viral/bacterial load, ammonia |
| Poultry red mite | Detection |
| Egg | Quality |
| Feed | Quality |
| Water | Quality |
| Light | Quantity and quality |
| Manure | Quality, such as manure water and nutrient content |
| Bones | Assessment of bone quality with MRI |
| Odor | Disease detection, Poultry red mite detection, fear |
| Robotics | Pick up eggs/manure/dead animals |
| Individual trackers | Measure activity, (nesting) behaviors, location (e.g. visits to free-range), movement between tiers |
| Worm | Detection, including pressure load |
| Cameras | Behavior in general, animal distribution, activity, posture, pecking behavior, grouping together, movement (through the aviary), (floor) egg laying behavior, toe pecking, restlessness, feather score, location (outdoor) , thermography (body temperature, chicken density), camera’s to check (dead hens on) egg belts |
| Sound | Pecking sounds, gakel calls/interspecies communication, loudness, frequency, frustration |
